# Intranasal administration of a two-dose adjuvanted multi-antigen TMV-subunit conjugate vaccine fully protects mice against *Francisella tularensis* LVS challenge

**DOI:** 10.1371/journal.pone.0194614

**Published:** 2018-04-23

**Authors:** Alison A. McCormick, Aisha Shakeel, Chris Yi, Hardeep Kaur, Ahd M. Mansour, Chandra Shekhar Bakshi

**Affiliations:** 1 Touro University California, College of Pharmacy, Vallejo, CA; 2 Department of Microbiology and Immunology, New York Medical College, Valhalla, NY; Midwestern University, UNITED STATES

## Abstract

Tularemia is a fatal human disease caused by *Francisella tularensis*, a Gram-negative encapsulated coccobacillus bacterium. Due to its low infectious dose, ease of aerosolized transmission, and lethal effects, the CDC lists *F*. *tularensis* as a Category A pathogen, the highest level for a potential biothreat agent. Previous vaccine studies have been conducted with live attenuated, inactivated, and subunit vaccines, which have achieved partial or full protection from *F*. *tularensis* live vaccine strain (LVS) challenge, but no vaccine has been approved for human use. We demonstrate the improved efficacy of a multi-antigen subunit vaccine by using Tobacco Mosaic virus (TMV) as an antigen carrier for the *F*. *tularensis* SchuS4 proteins DnaK, OmpA, SucB and Tul4 (DOST). The magnitude and quality of immune responses were compared after mice were immunized by subcutaneous or intranasal routes of administration with a TMV-DOST mixture, with or without four different adjuvants. Immune responses varied in magnitude and isotype profile, by antigen, by route of administration, and by protection in an *F*. *tularensis* LVS challenge model of disease. Interestingly, our analysis demonstrates an overwhelming IgG2 response to SucB after intranasal dosing, as well as a robust cellular response, which may account for the improved two-dose survival imparted by the tetravalent vaccine, compared to a previous study that tested efficacy of TMV-DOT. Our study provides evidence that potent humoral, cellular and mucosal immunity can be achieved by optimal antigen combination, delivery, adjuvant and appropriate route of administration, to improve vaccine potency and provide protection from pathogen challenge.

## Introduction

Tularemia is a fatal human disease caused by *Francisella tularensis*, a Gram-negative encapsulated coccobacillus bacterium [1, 2]. The pathogen itself can be carried by a variety of hosts and depending on the subspecies of bacterium and route of infection, the infectious dose is incredibly low of merely 10−10^3^ colony forming units (cfu) [1, 2]. *F*. *tularensis* is comprised of four main subspecies that vary in virulence and geographic location: *F*. *tularensis* subsp. *tularensis*, *holarctica*, *novicida* and *mediasiatica* [1–3]. Of these subspecies, the two most virulent subspecies of *F*. *tularensis*, Type A *F*. *tularensis* subsp. *tularensis* (SchuS4) and Type B *F*. *tularensis* subsp. *holarctica*, are most infectious and disease causing [1, 2, 4, 5]. The live vaccine strain (LVS) is derived from *F*. *tularensis* subsp. *holarctica*. The CDC lists *F*. *tularensis* as Tier 1 Category A select agent, classifying it at the highest level of potential biothreat agents due to its low infectious dose, ease of transmittance via aerosolized dissemination, and lethal effects [1, 5, 6]. To date, no vaccine has been licensed in the USA to prevent infection, so an ideal vaccine that offers safe, effective and long-lasting immunity in a short period of time is warranted [7].

Different approaches have been made in formulating vaccine against *F*. *tularensis*, including killed whole-cell, live attenuated and subunit vaccines [8]. However, no specific vaccine has been able to demonstrate safe and full immunity against virulent type A exposure, either due to poor protection, adverse effects, or potential residual virulence [1, 7–11]. Subunit vaccines are considered safer compared to live/inactivated vaccines because the focus is only on the immunologically relevant proteins of the pathogen [1, 2, 12, 13] that can be selected to induce strong cellular and humoral immune responses [12–14]. Although the single antigens can be selected from relevant serological studies can confer some immunity individually, single subunit vaccines are only partially protective [13, 15]. When tested against *F*. *tularensis* LVS in vaccinated mice, DnaK and Tul4 have enhanced both cellular and humoral immunity [16]. FopA, an outer membrane protein of *Francisella* has shown to offer full protection against LVS intranasal challenge and partial (80%) protection against intradermal LVS but has failed to protect against SchuS4 [17]. Outer Membrane Proteins (OMPs or Fop) are attractive vaccine target antigens due to their outer membrane cellular localization and easy access to antibodies [17, 18]. SucB (Dihydrolipoamide succinyltransferase) was also able to induce a humoral response in LVS-vaccinated mice [19]. Our hypothesis is that immunity against multiple antigens may be required to induce protective humoral and cellular immunity and that both arms of immunity are required for improved vaccine effectiveness.

Our previous study implemented a tri-valent subunit design, showing that a combination of antigens improved protection against tularemia using an efficient Tobacco Mosaic virus (TMV) based delivery platform [13]. Immunoreactive proteins DnaK, OmpA and Tul4 from the SchuS4 strain were conjugated to TMV and used to vaccinate mice intranasally, subcutaneously, or a combination of the two. Mice were then challenged with LVS, and various levels of protection were observed [13]. Although improved immunity was noted, complete protection was not achieved.

In order to optimize vaccine-induced protection, we compared the contributions of subcutaneous and intranasal vaccination on the potency of the immune response. We also added a fourth antigen, SucB, and several different adjuvants to enhance cellular and humoral responses. We tested the adjuvants Oligodeoxynucleotide 1826 (CpG), Polyinosinic-polycytidilic acid (Poly-IC or dIC), and MF59 by subcutaneous antigen delivery and CpG, dIC, and Cyclic diguanylate monophosphate (di-GMP) by intranasal delivery along with our TMV-conjugated vaccine. Our goal was to determine what effect adjuvant-vaccine combinations had on humoral and cellular immunity by either subcutaneous or intranasal administration. In the present study, we tested a multi-antigen subunit vaccine combining the *F*. *tularensis* proteins DnaK, OmpA, SucB and Tul4, while utilizing TMV as an antigen carrier. Administered with the optimal adjuvant and by altering route of administration we demonstrate augmented immunity that is both route and antigen specific. When challenged by *F*. *tularensis* LVS, the tetra-antigen, TMV-conjugated vaccine co-formulated with CpG or di-GMP adjuvant by intranasal route provides complete protection against lethal pathogen exposure. Our data provide evidence that potent humoral and cellular immune response can be induced quickly with optimal antigen selection and delivery, immune enhancers and appropriate route of administration, with long term immunity that can protect against pathogen challenge.

## Materials and methods

### Protein expression and purification and conjugation to TMV

Proteins were expressed by standard bacterial expression vectors, either in-house or provided by BioMatik (Canada). Protein purification was by metal affinity chromatography, and protein purity was assessed by SDS-PAGE. Concentration was determined by bicinchoninic acid assay (BCA; BioRad).

**Fig 1:**
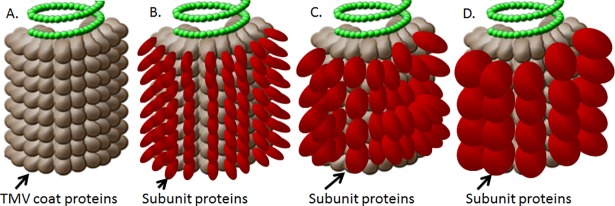
Subunit protein conjugation to the surface of TMV. A. A single TMV virion, consisting of a helical single RNA encapsidated by coat protein. B-D single TMV virion with the subunit antigen of increasingly larger sizes conjugated to the surface of the TMV rod.

Optimal conjugation conditions were determined for each protein as previously described, by conducting pilot studies [13]. The conditions are summarized in Table 1, similar to conditions used in a previous study [20]. Briefly, each protein was conjugated to TMV in a 1:1 molar ratio for Tul4 and a 1:1 mass ratio for larger proteins such as DnaK, OmpA, and SucB (to avoid steric hindrance at the TMV surface, as illustrated in Fig 1). Protein and TMV were combined at 3mg/ml final concentration in conjugation buffer (100 mM 2-(*N*-morpholino) ethanesulfonic acid + 500 mM NaCl solution at pH 6), Then, 1-ethyl-3-(3-dimethylaminopropyl) carbodiimide hydrochloride (EDC) was added at optimized concentrations, followed by N-hydroxysulfosuccinimide (NHS). Reactions were allowed to proceed for different time points, and then terminated by adding 1 mM methylamine. Final vaccine formulations were then dialyzed against PBS overnight to remove the reactive agents (EDC and NHS). SDS-PAGE was used to qualify conjugate characteristics, and optimized based on the time and concentration of EDC to generate minimal “free” protein antigen, which varied by protein (Table 1). The final dialyzed vaccine concentration was determined via BCA assay, and single use vaccine aliquots were frozen at -20°C until use.

**Table 1:** Optimal conjugation conditions for each antigen.

Antigen	Antigen:TMV (molar or mass ratio)	EDC	NHS	Total Reaction Time (hours)
Dnak	1:1 (mass ratio)	3 mM	5 mM	3
OmpA	1:1 (mass ratio)	6 mM	5 mM	3
SucB	1:1 (mass ratio)	3 mM	5 mM	2
Tul4	1:1 (molar ratio)	4 mM	5 mM	2

### Vaccination

Age-matched 40 day old female C57BL/6 mice were purchased from Charles River and housed at Touro University according to an IACUC-approved protocol, and under approved animal use guidelines. Mice were housed in standard cages with corn cob bedding, on a 12 hour light/dark cycle, with *ad lib* food and water, five mice per cage. Enrichment included cotton bedding and a domed plastic play/sleep enclosure. At 8 weeks of age, mice were randomly divided into groups of 5 mice (~20 grams), a sufficient number for measuring pilot immune response potency and quality prior to expanded studies in the challenge setting. For subcutaneous vaccine testing of TMV-DOST (a mixture of TMV-DnaK, TMV-OmpA, TMV-SucB and TMV-Tul4, delivering 10 μg of each antigen protein), alone (neat) or with dIC, CpG 1826, or “MF59” (AddaVax™; a squalene adjuvant most similar to the MF-59 formulation in human use), and PBS vaccinated mice were used as a negative control group (25 mice total). Because squalene adjuvant is not suitable for intranasal vaccination, mice were immunized with cyclic diguanylate monophosphate (di-GMP) as an alternative. For the intranasal study, mice were deeply anesthetized by intraperitoneal (IP) administration of a mixture of ketamine and xylazine until mice were unresponsive to a toe pinch. Each animal was administered 15 μL of vaccine in each nare. Animals were recovered on a warming pad until ambulatory (20 mice total).

Vaccinations were administered intranasally (i.n.) on day 1 and 35, where 20 μg of each TMV-conjugated antigen was combined with or without adjuvant, in PBS. For the adjuvant groups 2.5μg dIC, 20μg CpG, 1:1 ratio MF-59 with 100ul adjuvant, and 2.5μg di-GMP were used. All adjuvants were provided by InvivoGen.

Tail vein bleeds were performed and sera collected prior to the first vaccination (B = 0) and on days 14, 28 (post-vaccine 1), 49, and 63 (post-vaccine 2). Mice were briefly warmed and then tail vein blood was collected in microtainer tubes, centrifuged to separate the sera and then frozen until tested. Data from sera collected on days 28 and 63 are shown. No adverse events were observed that were specific for vaccine administration. At the time of spleen harvest for cellular analysis, or at the end of the study, mice were euthanized by approved methods.

### Immune response evaluation–antibodies

Mouse sera collected before and after each vaccination was analyzed for an IgG immune response by enzyme-linked immunosorbent assay (ELISA) as previously described [21]. Briefly, 96-well Nunc Immuno plates were coated with 5 μg/mL antigens (DnaK, OmpA, SucB, Tul4) in 50mM carbonate buffer (pH 9.6), at 4°C overnight. A titration of positive control mouse IgG (Southern Biotech) in carbonate buffer was used to standardize the antibody response quantitation across individual mice, groups and treatments. For isotype analysis, sera from all vaccinated animal groups was pooled and analyzed as a single sample, so relative ratios are shown rather than statistically significant differences between the groups.

Plates were washed three times in ELISA wash buffer (30mM NaCl + 0.1% Triton-X100) and incubated with ELISA block buffer (50mM Tris+ 0.05% Tween-20+ 2% BSA) for 1 hour at room temperature. A dilution series of sera was added for 1 hour at room temperature, washed and incubated in with HRP-conjugated secondary antibodies (IgG, IgG1, IgG2b, IgA; Southern Biotech) at 1:5000 dilution in PBS+2%BSA for 1 hour. After three final washes, and plates were developed with tetramethyl benzidine (TMB) HRP substrate, and stopped with 0.5N sulfuric acid. Plates were read on a microplate reader (Molecular Devices) at 450 nm and values are reported as relative IgG titer (in μg/ml) compared to an IgG standard curve. Baseline data (bleed 0, prior to immunization) was uniformly at the level of background (secondary only) for every animal as was also found for PBS vaccinated mice.

### Immune response evaluation–cellular responses

The number of Interferon-gamma (IFNγ) secreting cells, a relevant measure of the Cytotoxic T-lymphocyte (CTL) response, was tested by IFNγ ELISpot using single cell suspension of spleens from immunized mice, harvested on either day 65 or day 100 post-vaccine 1. Plates were coated with anti-IFNγ antibodies as per the manufacturers protocol (ELISpot Ready-SET-Go!;Affymetrix). Mouse splenocytes were isolated under aseptic conditions, counted for viable cells (Vicell XR, Beckman-Coulter), diluted and added as single cell suspensions of 1 or 2 × 10^5^ spleen cells/ml. Antigen specific responses were measured by stimulating with protein antigens (20 μg/ml), or Phorbol myristate acetate/Ionomycin (2.5 ng/ml, 250 ng/ml) as a positive control. Negative control cells were isolated from PBS immunized mice (Norm), and negative control stimulation (no stim) was with cells only. Cells were stimulated for 36 hours at 37°C, 5% CO_2_, 95% humidity, and then developed as per the manufacturer’s instructions. The reaction was stopped with distilled water, and plates left to dry overnight. Spots were counted with an AID ELISpot plate reader (minimum diameter, 40 μm). At day 100 post-vaccine 1, a recall response was measured by ELISpot for both IFNγ secretion, and by Granzyme B secretion (BD Biosciences), and by the same method of stimulation and analysis as for IFNγ.

### Pathogen challenge

**Fig 2:**
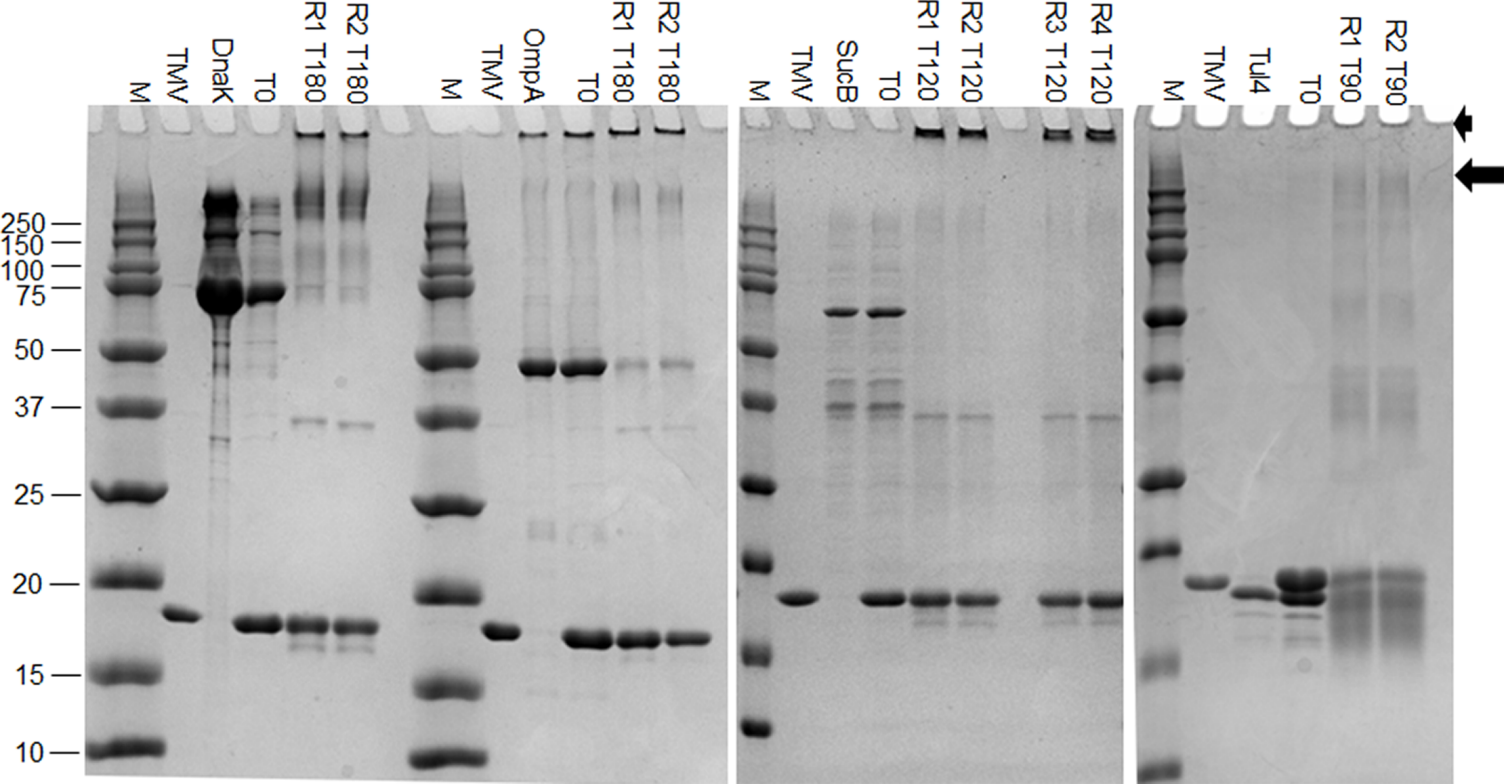
Antigen conjugation to TMV. Purified antigens were conjugated to TMV in a 1:1 molar ratio in an amide-carboxylate reaction using conditions optimized for each specific protein. M: Marker (Precision Plus Dual Color Standard- BioRad). T0: start time (mixture of TMV and antigen), R (1–4): independent reactions, T90-T180: time points (minutes) after reaction start. Successful conjugation is demonstrated by reduction in protein and an increase in conjugate product (>250kDa; arrow, or in the gel stack; arrowhead).

Age-matched 40 day old male and female mice (purchased from Charles River) were housed at New York Medical College according to an IACUC-approved protocol, and under approved animal use guidelines. Mice were housed in an area designed for infectious disease studies, where research staff received special training for care and handling of mice in a pathogen challenge setting. C57BL/6 mice (n = 7–10, 21–30 mice total) were vaccinated with 80 μg mixture of vaccine by intranasal dosing (10 μg of each antigen individually conjugated to 10 μg of TMV as shown in Fig 2) with 20 μg CpG or 2.5 μg diGMP. Pain and distress was minimized by performing all administrations in deeply anaesthetized mice that exhibited no toe-pinch reflex. Negative control mice were untreated. One prime vaccine given on day 1was followed by 1 booster vaccination at day 28. 21 days after the second vaccine (day 49 post-primary vaccination), mice were challenged with 1x10^5^ doses of LVS (10xLD_100_) and monitored daily for an additional 21 days for health, weight loss, and survival. Mice were euthanized immediately, by approved methods, when weight loss exceeded 25%. No mice died before reaching the criteria for euthanasia.

### Ethics statements

The use of animals and protocols were approved by the Institutional Animal Care and Use Committee (IACUC) of Touro University California (Protocol Number TUCA006AM01M-0519). This study was carried out in strict accordance with the recommendations and guidelines for care and use of animals. Animal experiments were conducted in the TUCA vivarium, which operates under OLAW assurance (A4510-01). Mice were subjected to either brief restraint for minor procedures, or general anesthesia using an anesthetic cocktail of ketamine (5 mg/kg) and xylazine (4 mg/kg) until unresponsive to a toe pinch. Anesthetized animals were recovered on a warming pad until ambulatory. All effort was made to minimize pain and suffering. Mice were euthanized at the end of the study by CO2 asphyxiation followed by cervical dislocation, as recommended by the Panel on Euthanasia of the American Veterinary Medical Association, and according to IACUC protocol. Although no formal toxicology analysis was completed, mice recovered completely from all immunization protocols, without signs of distress, regardless of adjuvant co-formulation.

The use of animals and protocols were approved by the Institutional Animal Care and Use Committee (IACUC) of New York Medical College (Protocol Number 22-2-0416H). This study was carried out in strict accordance with the recommendations and guidelines of National Council for Research (NCR) for care and use of animals. All the animal experiments were conducted in the centralized Animal Resources Facilities of New York Medical College licensed by the USDA and the NYS Department of Health, Division of Laboratories and Research and accredited by the American Association for the Accreditation of Laboratory Care. Mice were administered an anesthetic cocktail consisting of ketamine (5 mg/kg) and xylazine (4 mg/kg) and underwent experimental manipulation only after they failed to exhibit a toe pinch reflex. Mice exhibiting more than 25% weight loss, anorexia, dehydration and impairment of mobility were removed from the study and euthanized by approved means. Humane endpoints were also necessary for mice which survived at the conclusion of the experiment. Mice were administered an anesthetic cocktail of ketamine and xylazine intraperitoneally and then euthanized via cervical dislocation followed by cardiac puncture, a method that is consistent with recommendations of the Panel on Euthanasia of the American Veterinary Medical Association. In all experimental procedures, efforts were made to minimize pain and suffering.

### Statistical analysis

Statistical significance was determined via unpaired non-parametric t-test or Kaplan-Meier Log Rank test in GraphPad Prism, where p-values ≤0.05 were considered statistically significant.

## Results

### Conjugation of *Ft* SchuS4 antigens to TMV

Single protein antigens were chemically conjugated to TMV 1295.10, which expresses a modified coat protein with a surface-exposed lysine group that allows amide-directed chemical conjugation to the carboxyl group of the antigen [22]. Fig 1A shows a cartoon of the TMV virion prior to- and after the virion is conjugated to an antigen of size equal or less than coat protein (Fig 1B), or with antigens of increasing molecular weights (Fig 1C, Fig 1D). Not all coat proteins will react with the subunit protein directly, when molecular weight causes steric hindrance at the virus surface.

Conjugation reaction of protein mixed with a 1:1 molar ratio of TMV, and catalyzed with EDC and NHS which covalently links amide to carboxyl groups, resulting in an amine final linkage. Optimal conditions for TMV:protein molar ratio, length of reaction, and EDC concentration were determined in previous studies and small pilot studies, as each protein reacts differently. Optimal reaction condition used the lowest concentration of EDC and NHS, and shortest reaction time period to avoid over-conjugating the antigen to the coat proteins. Table 1 presents the reaction conditions optimized for each antigen.

Reactions were analyzed by SDS-PAGE against the unconjugated antigen and unconjugated TMV starting material, to confirm that pilot scale reaction conditions were repeatable, with a minimum of free antigen protein. Fig 2 shows the scale up conjugation reproducibility, with conjugate products visible as a high molecular weight band greater than 250 kDa (short arrow), and accumulating in the gel/stack interface (arrowhead). The final conjugates were dialyzed in PBS and analyzed via BCA assay to determine final vaccine concentration.

### Immune response evaluation—Antibodies

**Fig 3:**
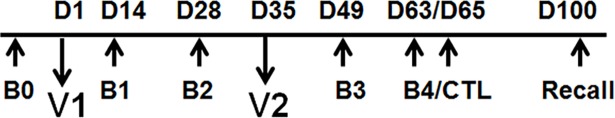
Timeline for immunization, serum and spleen collection. Vaccinations occurred on days 1 and 35, with serum collection on days 0, 28, 49 and 63. Spleen harvest for IFNγ and Granzyme B analysis occurred on days 65 and 100 post-primary vaccination.

To test the immune response conferred by these vaccines as a multi-antigen formulation, we combined each TMV-antigen at 10 μg antigen dose (20 μg TMV-antigen) to create an 80 μg TMV-DOST mixture. We used this mixture to vaccinate mice by subcutaneous and intranasal routes of administration. 8 week old C57BL/6 mice were immunized twice (day 1 and day 35) with the TMV-DOST vaccine with or without an adjuvant. The schedule of vaccination, bleeds and spleen harvest for cellular analysis is shown in Fig 3.

By subcutaneous route of administration, we tested adjuvants dIC (a TLR3 activator), CpG (a TLR9 activator), and MF59 (a broadly effective adjuvant) as described in Methods. In the intranasal dosing studies, we replaced MF59 because it is not a suitable adjuvant for i.n. route of administration [23]. Instead, we tested di-GMP, which has shown to be effective by the i.n. route [24]. Serum was collected by tail vein bleeds prior to the first vaccine (b0; baseline immunogenicity), and after each vaccine on days 14, 28 (after the first vaccine) and days 49 and 63 (after the second vaccine). Cellular analysis was completed on days 65 and 100.

**Fig 4:**
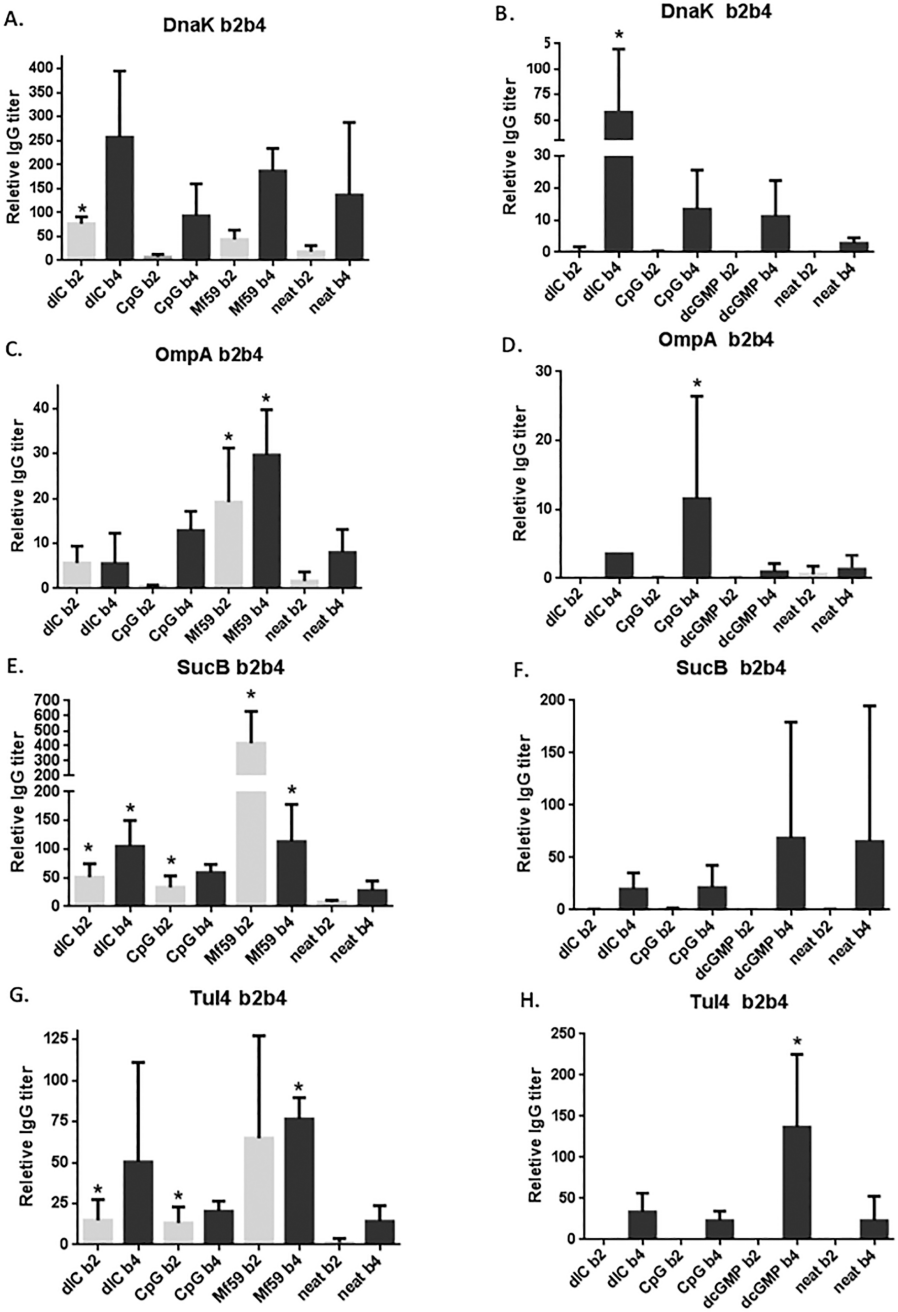
Antigen-specific IgG titers post-vaccine 1 and 2 after TMV-DOST vaccination. C57BL/6 mice were used in a subcutaneous (n = 4) and intranasal (n = 5) immunization with 20μg each TMV-conjugated tetravalent vaccine with or without (neat) adjuvant on day 1 (vaccine 1) and day 35 (vaccine 2). Sera was collected on day 28 (bleed 2) and day 63 (bleed 4) and tested for total IgG by ELISA on plates coated with 5μg of respective protein and quantified against a standard curve of antigen specific antibody. Post-vaccine 1 bleed 2 (b2) and post-vaccine 2 bleed 4 (b4) data is shown for Dnak (A), OmpA (B), SucB (C), and Tul4 (D). Means are presented ± standard deviation, where statistical significance is p<0.001 (*) compared to vaccination without adjuvant, using a nonparametric t-test.

Sera were analyzed via ELISA for antigen-specific IgG antibody levels. Fig 4 shows the relative antibody titers for each antigen from bleeds 2 (b2: day 28 post-vaccine 1) and 4 (b4: day 35post-vaccine 2). As demonstrated in Fig 4, responses are antigen and adjuvant-dependent and the second vaccination improved the immune response in most cases. Subcutaneous immunization (Fig 4A, 4C, 4E and 4G) had a higher IgG response over i.n. dosing (Fig 4B, 4D, 4F and 4H) across all experiments. For DnaK, dIC conferred the best response in both subcutaneous (Fig 4A) and i.n. (Fig 4B) route of administration. For OmpA, Mf59 significantly improved immune response compared to the un-adjuvanted vaccine (neat; *p<0.05) after subcutaneous administration, where IgG levels after both the first and second vaccine were statistically significant (Fig 4C). However, by i.n. administration CpG conferred the highest antibody response after the second vaccine only, where response was low or immeasurable after the first vaccine (Fig 4D). For SucB, after the first subcutaneous dose all adjuvants significantly improved IgG titers compared to neat vaccine (Fig 4E). After the second dose, only dIC and MF59 improved IgG titers. On the other hand, adjuvant was not necessary to confer a potent immune response against SucB after intranasal dosing; the neat vaccine performed well (Fig 4F). Out of all the antigens, Tul4 showed most similar titers between the two routes of administration, and only differed by adjuvant choice (Fig 4G and 4H). MF59 worked best in the subcutaneous group, especially in inducing a strong first dose response, one that did not increase significantly after the second vaccine (Fig 4G). After i.n. dosing, di-GMP induced the highest IgG titer against Tul4 (Fig 4H). For all antigens given by the i.n. route of administration, boosting with a second dose was required to induce immunity.

**Fig 5:**
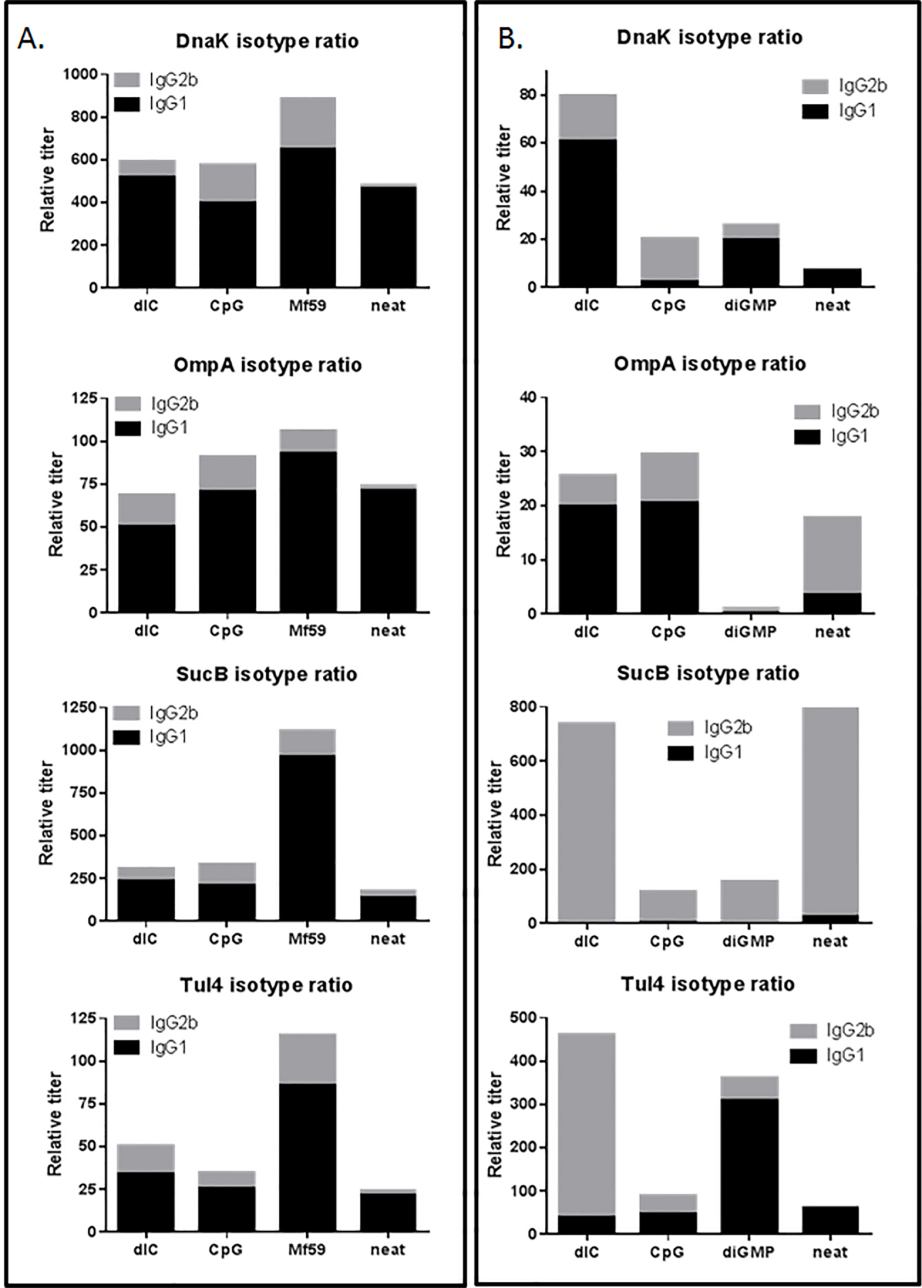
IgG isotype profile analysis of TMV-DOST (D, DnaK;O, OmpA; S, SucB; T, Tul4) vaccinated mice. Bleed 4 sera pooled from mice after the second immunization of 20 μg TMV-conjugated DOST antigen vaccine with (dIC, CpG, MF59 or diGMP) or without (neat) adjuvant was analyzed by ELISA against each antigen-using isotype specific IgG1 and IgG2b secondary antibodies to deduce type of immune response. Isotype profile is depicted as a ratio of the two, where direct addition gives total isotype content. **A**: Subcutaneous immunization profile. **B**: Intranasal immunization profile.

IgG titers reflect the overall magnitude of the antigen-specific antibody response, but do not adequately describe the quality of the immune response. The ratio of IgG1 to IgG2 is a strong indicator of T-cell help during B-cell activation with higher IgG2 titers reflecting the presence of Th1 cytokines like IFNγ during B-cell activation, which is known to be important for pathogen killing, especially for an intracellular pathogen like *F*. *tularensis* [25, 26]. We measured IgG1 and IgG2 isotypes in pooled bleed 4 sera from each vaccine group, and Fig 5 shows the IgG2b:IgG1 ratio determined from this analysis. By subcutaneous administration (Fig 5A), adjuvants greatly enhanced the IgG2 content of the immune response profile. Neat groups were primarily IgG1-dominated, with little or no IgG2 present. MF59 conferred the greatest total response across all antigens, while IgG1 content of the neat groups were comparable to the other two adjuvants dIC and CpG.

Although the subcutaneous immunization showed similar patterns of immune response activation, the i.n. immunization responses (Fig 5B) were varied significantly by antigen and adjuvant. IgG2 content was most enhanced by adjuvant for DnaK and Tul4 compared to neat (unadjuvanted) vaccine, which induced little to no IgG2 isotype titers. DnaK dIC also had a boosted IgG1 content compared to other vaccines in that group. For OmpA, adjuvant addition only improved IgG1 titers when compared to neat vaccination, and diGMP significantly reduced both isotype responses. Interestingly, SucB had a predominant IgG2 isotype with little or no IgG1 response irrespective of adjuvant, unlike all other groups in this analysis, and responses were not boosted by any adjuvant co-administration. Tul4 dIC group showed the most striking improvement in IgG2 levels, and Tul4 diGMP showed improvement in both IgG1 and IgG2response magnitude.

**Fig 6:**
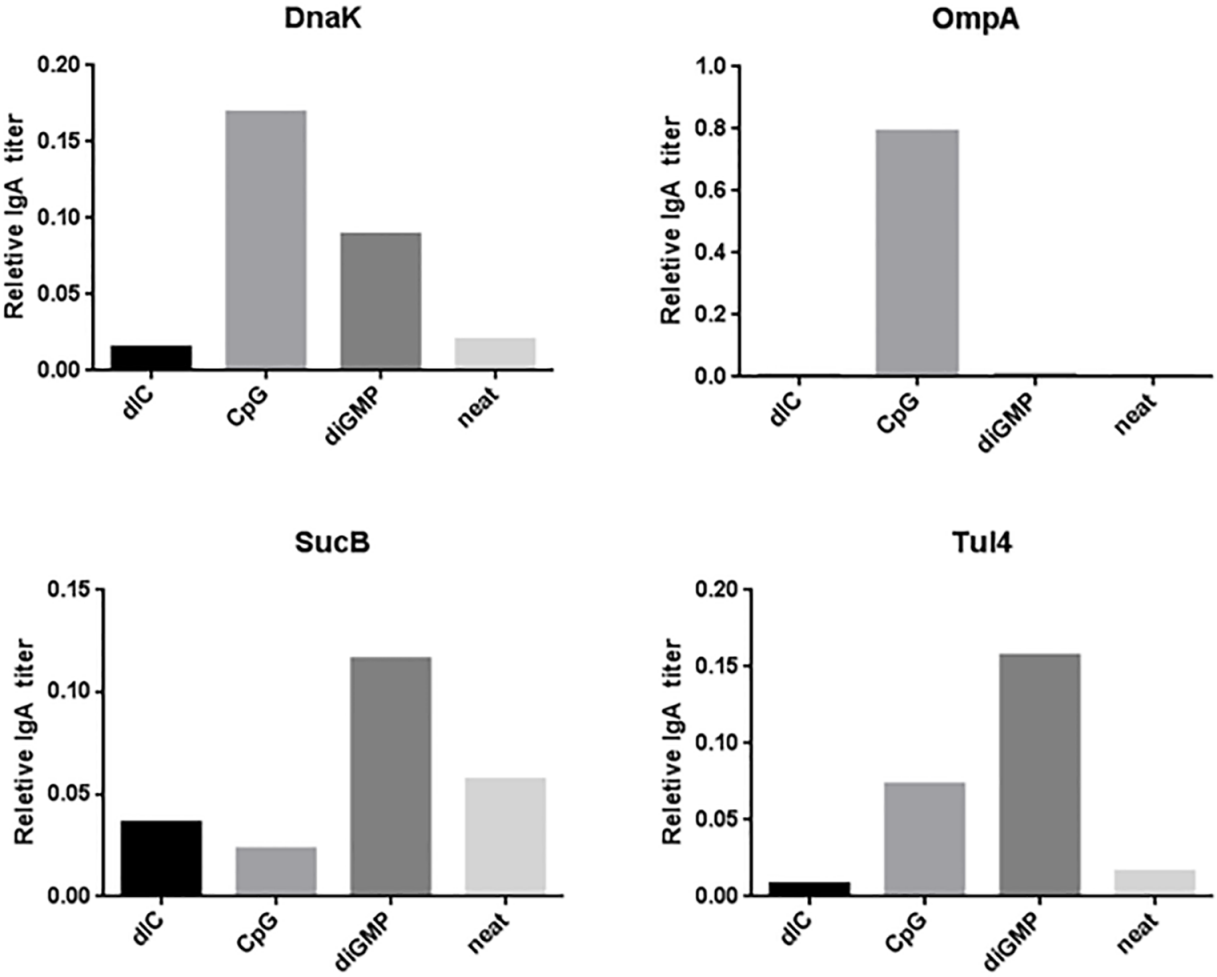
Systemic IgA response to TMV-DOST intranasal vaccination. Systemic serum IgA titers against each specific antigen in the pooled sera of mice immunized twice intranasally with the TMV-conjugated tetravalent vaccine as described in Fig 5.

In addition to IgG1 and IgG2b response analysis, systemic IgA response was analyzed in the pooled B4 sera of i.n. immunized mice. IgA is an indicator of mucosal immunity, which is relevant to the i.n. route of pathogen administration and the most potentially lethal route of infection for *F*. *tularensis*. Fig 6 shows that systemic IgA can be measured in response to mucosal vaccination, and the response varied by antigen. CpG conferred the highest IgA titer for DnaK (Fig 6A), followed by diGMP, whereas dIC and neat (no adjuvant) were similar in response. OmpA CpG induced the only mucosal immunity (Fig 6B), and no responses were seen in any other group. diGMP conferred the highest IgA titers in both SucB (Fig 6C) and Tul4 (Fig 6D), with low but measurable responses in neat and other adjuvant groups.

### Cellular immune response—Evaluation by IFNγ ELISpot

**Fig 7:**
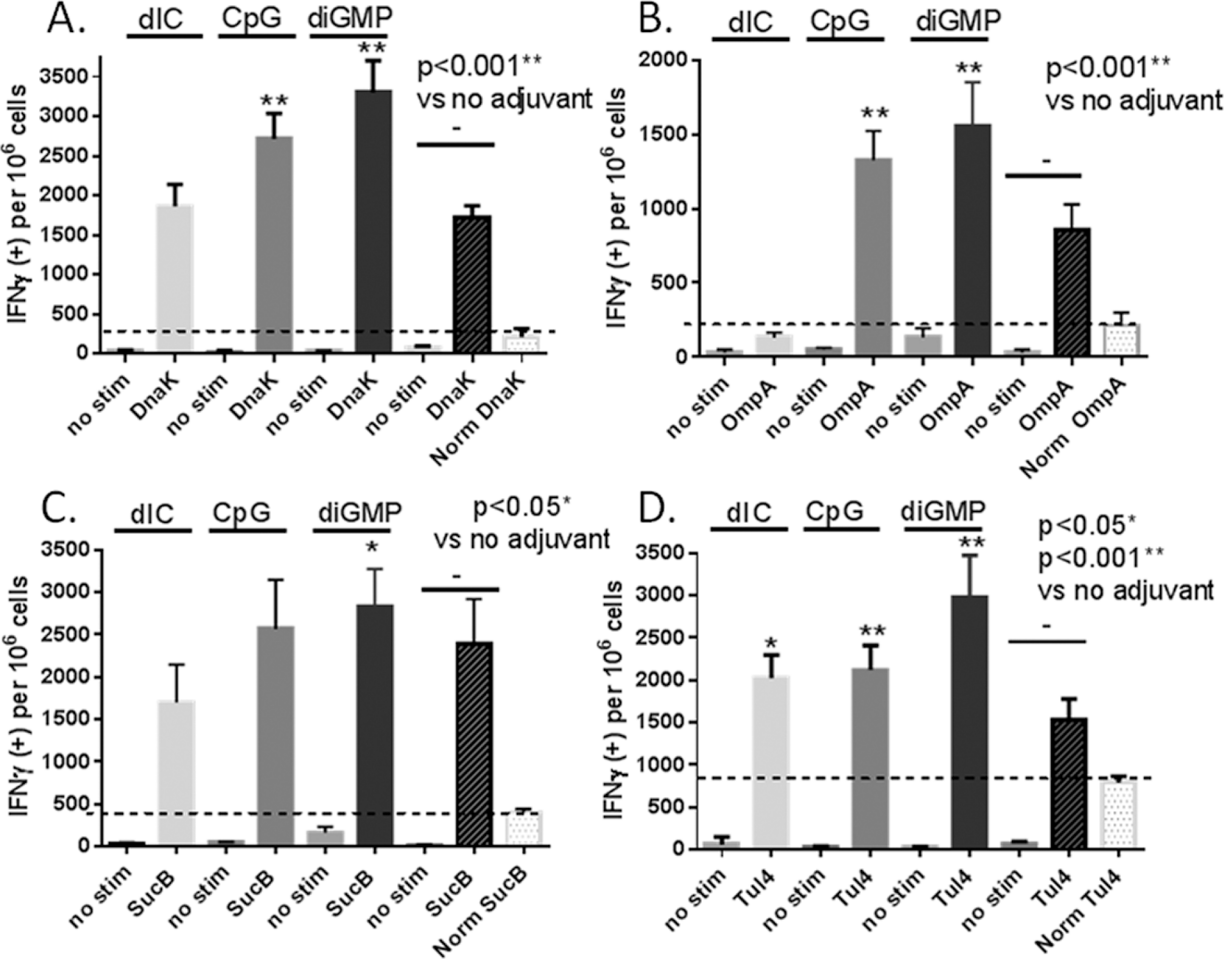
IFNγ ELISpot assay on splenocytes of mice vaccinated two times with TMV-DOST vaccine. Splenocytes were isolated from 2 mice per group on D65 after intranasal vaccination and incubated on IFNγ antibody coated plates at a final concentration of 10^5^ cells/well. Whole protein antigen stimulants were added at 20 μg/mL, and plates incubated at 37°C in a 5% CO_2_, 99% humidity incubator for ~36h. IFNγ secreting cells were detected and developed using Mouse IFNγELISpot Ready-SET-Go! Kit, developed with AEC, and read on an AID ELISpot plate reader. IFNγ secretion from spleen cells stimulated with DnaK (**A**.) OmpA (**B.**) SucB (**C.**) and Tul4 (**D**.) are compared to no stimulation (no stim), or PBS vaccinated mice (Norm). Values were adjusted positive IFNγ spots per 10^6^ cells, and groups that show significant augmentation with adjuvants compared to no adjuvant are shown (*p<0.05, **p<0.001). Means are presented ± standard deviation, and p values were calculated by non-parametric t-test. Results are representative of two experiments.

In addition to measuring humoral immunity by IgG and isotype titers, we also measured cell-mediated immunity by IFNγ secretion assay (IFNγ ELISpot Fig 7). Splenocytes from mice dosed by i.n. immunization were sacrificed (2 mice per group), spleens were removed, and single cell suspensions were tested for the presence of IFNγ secreting cells after antigen stimulation. Little or no IFNγ secreting cells were measured in the absence of stimulation (Fig 7; no stim). Even though the PBS vaccinated mouse control (Norm) showed some immune response to the antigen stimulation, all vaccine groups had significantly improved antigen-specific cellular responses compared to PBS (p>0.001). OmpA (Fig 7B) induced the lowest unadjuvanted response, and SucB (Fig 7C) induced the highest overall response without adjuvant, suggesting that SucB may be more innately immune-stimulatory. Both DnaK (Fig 7A) and Tul4 (Fig 7D) induced the highest levels of IFNγ secretion when vaccines were administered with diGMP, with values exceeding 3000 spot forming units (SFU) per million cells. Notably, IFNγ secretion was robust (>1000 SFU) independent of adjuvant formulation, but responses were significantly boosted with adjuvant.

Out of all the adjuvant groups, dIC adjuvant vaccine group was either no better than the no adjuvant group (DnaK;Fig 7A) or induced lower IFNγ production per 10^6^ CTL cells when compared to the no adjuvant group (OmpA and SucB). CpG vaccine groups induced statistically significant increases in CTL responses against TMV-DOT antigens (**p<0.001), with di-GMP conferring the strongest cell-mediated immune response for all four TMV-DOST antigens (**p<0.001). This is in contrast to the IgG isotype response, where dIC induced the best IgG2/1 ratio for both SucB and Tul4. Overall, the magnitude of the IFNγ response (1500–3000 SFU per million cells) was far greater than observed for other TMV-subunit antigen vaccine formulations tested in our lab (personal observation), most likely due to i.n. route of administration.

### Pathogen challenge

**Fig 8:**
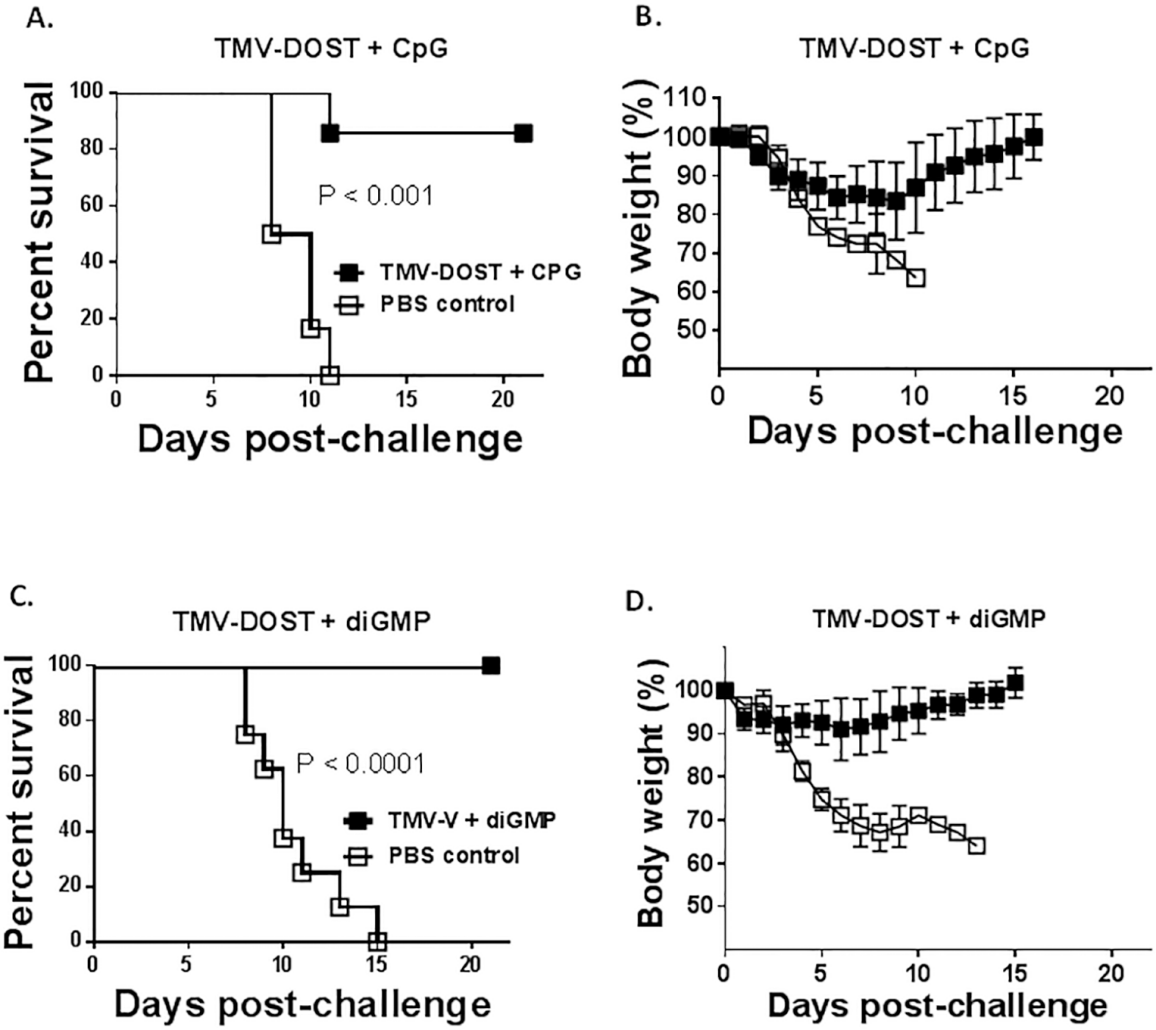
Pathogen challenge with *F*. *tularensis* LVS. C57BL/6 mice (n = 7–10) were vaccinated 2 times, 28 days apart, with either TMV-DOST with adjuvant or with PBS + adjuvant. Mice were challenged with 10xLD_100_ dose of *F*. *tularensis* LVS 21 days after the second vaccine and monitored for weight loss and survival. Mice were euthanized when weight loss exceeded 30%. (**A.**) Mice were vaccinated with TMV-DOST vaccine + CPG, or left untreated, and survival and body weights were recorded (**B.**) for 21 days. TMV-DOST was administered with di-GMP or left untreated and survival (**C.**) and body weights (**D.**) were recorded for 21 days. Statistical significance was determined via Kaplan-Meier Log-rank Test (GraphPad Prism).

Although immunogenicity analyses for humoral and cellular immunity are helpful in deducing an immune response profile of each vaccine formulation group, the combined effect must be tested against pathogen challenge to determine if vaccine is ultimately protective. Fig 8 compares the survival curves of mice immunized with 2 intranasal vaccinations 28 days apart with TMV-DOST tetravalent vaccine given with the two adjuvants that promoted the highest cellular activation profiles, CpG (Fig 8A and 8B) or diGMP (Fig 8C and 8D). Mice were challenged with 10 times the LD_100_ dose of LVS infection 21 days post-vaccine 2. Both TMV-DOST vaccine groups were significantly protected compared to PBS controls (p<0.001). TMV-DOST vaccine given with CpG adjuvant protected 85% of mice (A), with minimal weight loss (B) that recovered during the challenge interval. TMV-DOST vaccine given with diGMP adjuvant was completely protective, with little or no weight loss during the challenge interval. Pathogen challenge of untreated control mice was uniformly fatal.

### Granzyme B and IFNγ recall response

**Fig 9:**
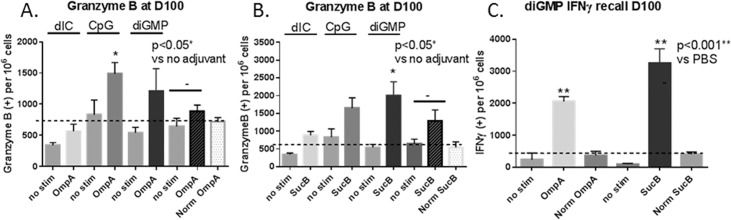
Recall IFNγ ELISpot and Granzyme B cellular immune response on D100. **A.** Splenocytes of twice-vaccinated mice were harvested at Day 100 after intranasal vaccination as described in Fig 7. (**A.**) Splenocytes from TMV-DOST vaccine groups were tested for Granzyme B secretion after antigen stimulation by the low responder (OmpA) and (**B.**) high responder (SucB) antigens given with no adjuvant (-), dIC, CpG or diGMP adjuvant. (**C**.) IFNγ responses after TMV-DOST + diGMP are compared to no stimulation, or PBS vaccinated mice (Norm) groups (2 mice per group). Values for both Granzyme B and IFNγ were adjusted positive spots per 10^6^ cells, and groups that show significant augmentation with adjuvants compared to no adjuvant are shown (** p<0.001 and *p<0.05). Means are presented ± standard deviation, and statistical analysis was done via non-parametric t-test.

In order to test the durability and T cell activation potential of the TMV-DOST multiantigen vaccine, spleen cells from animals vaccinated as in Fig 7 were analyzed at day 100 post vaccine 1 by ELISpot for Granzyme B secretion (Fig 9) after stimulation with OmpA (lowest unadjuvanted responder; Fig 9A) and SucB (highest unadjuvanted responder; Fig 9B). Granzyme B is a marker for CTL and NK cell activation, with the potential to kill *F*. *tularensis* infected cells by caspase-mediated apoptosis [27]. Mice immunized with the vaccine + di-GMP stimulated the highest Granzyme B response to SucB stimulation, whereas the OmpA group had the highest stimulation with when the vaccine was adjuvanted with CpG (p<0.05) compared to unadjuvanted vaccine groups. Overall, SucB stimulated responses were higher than for OmpA, as also seen for IFNγ secretion. Spleen cells were also tested by ELISpot for IFNγ secretion after OmpA and SucB stimulation for just the diGMP group (Fig 9C), and positive SFU cell continued to increase compared to analysis at D65 (Fig 7).

## Discussion

This study stems from the idea that developing a successful subunit vaccine that can protect against pathogenic challenge requires: 1) a combination of antigens that is sufficient to confer effective protection; 2) a method to successfully deliver antigen and improve presentation; 3) immune enhancers that can produce a more robust and durable immune response than antigen alone; and 4) route of administration that is relevant to pathogenesis that can rapidly confer optimized immunity and protection. Our results validate our hypothesis, that a multi-antigen TMV subunit vaccine can provide full protection against *F*. *tularensis* LVS challenge with just two doses of vaccine. This outcome is achieved by selecting four immune activating antigens, utilizing TMV as a subunit protein antigen carrier that induces optimal antigen presentation, adding adjuvants and administering vaccines by i.n. administration.

Previously, our study formulated a tri-antigen subunit vaccine against tularemia by including three *F*. *tularensis* proteins DnaK, OmpA, and Tul4, conjugated to an efficient TMV-based delivery platform. Mice were immunized via i.n. route or a combination of i.n. and subcutaneous routes, and challenged with *F*. *tularensis* LVS. Although the study reported an improved immunity, it provided only 60% protection against LVS challenge [13]. We hypothesized that complete protection may be achieved with the addition of another antigen (SucB) and adjuvants that will boost the magnitude or quality of immune response. SucB has been selected because it is 1 of 5 antigens recognized by human sera and 1 of 20 antigens in the immune sera of LVS-vaccinated mice [18, 19, 28]. Additionally, a previous study used a SucB antigen vaccine to provide partial protection against another bacterial pathogen *Brucella abortus* [29]. This study showed that SucB induced a humoral response in vaccinated mice with isotype switching and cell-mediated immunity that was confirmed by cytokine profiling and lymphocyte proliferation assays [29]. In our current study, we formulated TMV vaccines with DnaK, OmpA, Tul4, and SucB, (TMV-DOST) where each antigen is chemically conjugated to TMV separately and then mixed together as a multi-antigen conjugate vaccine (TMV-DOST) composed of equal amounts of subunit vaccine protein and TMV (Fig 2).

Mice were immunized with the TMV-DOST vaccine, with or without adjuvants dIC, CpG, MF59, by the subcutaneous route, or with dIC, CpG, di-GMP by intranasal dosing. MF59, a squalene oil-in-water emulsion, acts as a depot adjuvant. MF59 increases Th1 responses (related to cellular immunity), but confers a stronger Th2 response leading to antibody production and humoral immunity [30]. CpG has been shown to increase production of antigen-specific antibodies and CTL responses in murine models, acting via the TLR9 pathway which is synergistic with the anti-viral TLR7 pathway [31, 32]. The CpG used in our study is B-class, stimulating murine B-cell activity and Th1 cells which in turn promote dendritic cell maturation and cytotoxic T-cell activity [33, 34]. Similarly, dIC is a TLR3 adjuvant and induces Type I Interferon expression, stimulating recruitment and activation of macrophages and dendritic cells that promote both Th1 and Th2 immunity, which leads to B-cell and helper T-cell production [34], especially in the presence of particulate antigens like TMV-DOST [35]. Adjuvant di-GMP activates STING signaling, stimulating Type I Interferon expression. These classes of adjuvants should be synergistic with anti-viral responses induced by the single-strand RNA in TMV, which putatively acts through TLR7 [36].

As demonstrated in Fig 4, all antigens stimulated single dose antigen-specific IgG antibody titers that are antigen and adjuvant dependent, with improved immune response with second vaccination in most cases. Across all experiments, subcutaneous dosing (Fig 4A, 4C, 4E and 4G) had higher IgG response compared to intranasal dosing (Fig 4B, 4D, 4F and 4H). Although intranasal IgG responses were lower, intranasal immunization showed potential for improved quality of humoral and cellular immune response based on results from isotype analysis (Fig 5 and Fig 6) and CTL responses (Fig 7 and Fig 9). Intranasal dosing conferred a better isotype response; not only were IgG1 titers measurable in most cases (Fig 5B), there was heightened class-switching to IgG2 antibodies when compared to subcutaneous dosing results (Fig 5A), similar to other studies [37]. In general, IgG2 antibodies have the potential to provide enhanced pathogen clearance and protection because Fc receptor activation stimulates macrophages and killer T-cells [13, 38]., and in previous studies, improved protection was observed after adoptive transfer of primarily Ig2 subclass of antibodies in a rat SchuS4 challenge model [25]. Unexpectedly, SucB induced a strongly dominant IgG2 isotype irrespective of adjuvants (Fig 5F), unlike other groups in the analysis. This confirms a promising subunit antigen vaccine candidate against *F*. *tularensis* and warrants further analysis.

Secondly, by intranasal administration, TMV-DOST stimulates robust IFNγ secretion by ELISpot (Fig 7 and Fig 9) that was significantly intensified (at levels more than 2x higher) with addition of adjuvant. TMV-DOST CpG and TMV-DOST di-GMP vaccine groups induced statistically significant increases in CTL responses, with di-GMP conferring the strongest cell-mediated immune response at day65 post-vaccine 1. We then tested for a recall IFNγ response at day 100 for the diGMP group. Stimulation with either OmpA (lowest unadjuvanted responder) or SucB (highest unadjuvanted responder) induced IFNγ response levels greater than measured at day65, suggesting robust recall responses. IFNγ responses were shown to be critically important in providing protection after intranasal inactivated LVS vaccination [39], and improved gender-specific survival in male mice [40], We believe that these heightened CTL responses to subunit proteins are a result of TMV conjugation, which likely improved antigen delivery to mucosal tissue antigen retention on mucosal surfaces, subsequent recruitment of APCs, and the stimulation of TLR-mediated immunity to the plus-strand single-stranded RNA in the TMV virus, all characteristics known to improve mucosal immunity [41].

In this study, we demonstrate the intranasal route of administration is a key contributor to improved pathogen clearance and protection compared to our previous study. Vaccination at the site of pathogen entry has been known to offer superior protection against respiratory disease including plague, measles and influenza [37, 42–45]. Therefore, a vaccine that elicits a mucosal as well as systemic immunity could provide superior protection. A study by Arnaboldi *et*. *al*., demonstrated that TMV-based subunit vaccine, when administered intranasally, was able to induce complete protection against the pneumonic plague without adjuvant [46]. These results confirm the utility of TMV-Ag intranasal vaccine efficacy, since our study utilized the same route of administration and TMV delivery platform to induce a quality immune response.

Intranasal dosing of our vaccine promoted systemic IgA antibody production (Fig 6). Although the response was of low titer compared to IgG, we suspect that different sample collection methods might measure a higher response. For example, using nasal washes instead of sera collection from tail vein bleeds might reflect a higher IgA response overall. Furthermore, intranasal dosing has also been shown to induce immunity at distant mucosal sites, such as the gut, which can be sampled by fecal IgA. Intranasal vaccination against the gut pathogen norovirus showed protection against gastroenteritis and other disease complications as well as production of dose-dependent IgG and IgA memory B-cells in peripheral blood [47, 48]. This suggests that intranasal vaccines can confer both mucosal and systemic protection, as has been seen by other groups after intranasal dosing of inactivated LVS given with Ctb as an adjuvant [39]. Our results prove that intranasal dosing stimulated low but measurable systemic immunity by the presence of IgA antibody titers by intranasal vaccination (Fig 4), alluding to the possibility of mucosal immunity at distant sites as well. IgA responses have been shown to be important in inducing partially protective mucosal immunity against inactivated LVS after intranasal vaccination with IL-12 adjuvant [49]. Increased LVS susceptibility in IgA-/- mice [50] also indicates an important role of IgA in native immunity after respiratory pathogen exposure.

Intranasal vaccination has been a challenge because of poor efficacy, so it was important in this study to select a formulation, antigens and adjuvants that overcome barriers to mucosal immunity [41]. These include the poor permeability of the vestibular area of the nasal cavity, hair and mucus barriers to absorption, rapid mucosal turnover limiting vaccine retention time, and the nasal environment including enzymes and pH [41, 51]. These considerations were applied to our study, where the particulate quality of the TMV-DOST vaccine paired with the immunological properties of CpG and di-GMP yielded durable antibody and cellular responses with immunity that also rapidly confers protection against pathogen challenge.

Numerous single or bi-valent antigens have been tested as potential vaccine formulations with limited success due to partial protection against *F*. *tularensis* LVS challenges [16, 17, 52]. Hickey *et*. *al*., formulated a single antigen vaccine using recombinant OmpA (or FopA) incorporated in liposomes in conjunction with IL-12 and aluminum hydroxide. Mice were vaccinated heterogeneously (s.c./i.p. and i.n. booster) and pathogen challenge was done using both *F*. *tularensis* LVS and SchuS4 strain. Although the single antigen vaccine conferred mixed protection from *F*. *tularensis* LVS challenge (80% survival from lethal i.d. challenge and 100% survival from lethal i.n. challenge), it failed to protect against SchuS4 [17]. Kaur *et*. *al*., tested an adenovirus-based Tul4 vaccine that improved protection compared to recombinant Tul4 against i.p. *F*. *tularensis* LVS challenge, but it failed to provide complete protection (60% survival by i.m. vaccination and 20% survival by i.d. vaccination)[52]. Ashtekar *et*. *al*., created a bi-valent vaccine using DnaK and Tul4, and after demonstrating immunogenicity of the two antigens, thrice intranasal vaccinated mice were challenged with low and high doses of LVS. Again, the subunit vaccine improved survival but did not offer full protection with only 80% and 35% survival for the low and high LVS infections, respectively [16]. In the case of complex pathogens like *F*. *tularensis*, a single or dual antigen vaccine was not sufficient in conferring protective immunity, irrespective of delivery method. Our data show that both CpG and diC-GMP adjuvants combined with a multi-subunit vaccine can improve protection by inducing immune responses against multiple antigenic proteins after just two vaccinations. Our tetravalent TMV-DOST vaccine was able to elicit full protection against *F*. *tularensis* LVS challenge with diGMP adjuvant.

Although our TMV-DOST+diGMP multi-antigen vaccine gave full protection against pathogenic *F*. *tularensis* LVS challenge, it is critical to provide protection against the more virulent type A, SchuS4 strain. Various researches have been conducted with live attenuated, inactivated, and subunit vaccines, however full protection against SchuS4 remains unreported [17, 26, 38, 53–57]. Recently, Richard *et*. *al*., were able induce about 60% protection against intranasal SchuS4 challenge by incorporating lysates from partially attenuated *F*. *tularensis* LVS strains into catanionic surfactant vesicle nanoparticles (LVS-V), with monophosphoryl lipid A (MPL) adjuvant, administered via intraperitoneal/intranasal routes [26]. Similarly, Huntley *et*. *al*., study showed about 50% protection against intranasal SchuS4 challenge using an extraction of native outer membrane protein (OMP) given with Freund’s adjuvant, administered intraperitoneally [53]. The latter study demonstrated OMPs can serve as a partially protective vaccine against respiratory challenge with SchuS4 strain and warranted the use of immune-stimulating complexes and adjuvants in future studies [53, 58]. It should be noted that our study includes an outer membrane protein (OmpA) and have included adjuvants to provide complete protection in *F*. *tularensis* LVS challenges. However, our interpretation of the data suggests that SucB may be more inherently antigenic than OmpA, and its inclusion provided superior immune protection when added to a vaccine cocktail that also included OmpA. Further testing will be required to determine if our TMV-DOST vaccine will be able to induce better protection against SchuS4 challenge, compared to other studies testing for protection after subunit vaccination.

### Conclusions

Our data shows that a multi-antigen subunit vaccine using the antigens DnaK, OmpA, SucB, and Tul4 conjugated to TMV rapidly induces potent immune responses in mice by either subcutaneous or intranasal administration. Furthermore, this response is augmented with the addition of adjuvants that enhance the immune response profile, either magnitude (s.c.) or quality (i.n.). Not only does our vaccine confer humoral immunity and isotype class switching, it also induces durable cellular immunity, including both IFNγ and Granzyme B secretion after antigen specific stimulation. Intranasal dosing with TMV-DOST with either CpG or diGMP was able to induce protective immunity against LVS pathogen challenge. Our current data suggests the potential for protection against pathogen challenge with the more virulent Type A, SchuS4 strain.
